# Transferrin Identification in Sterlet (*Acipenser ruthenus*) Reproductive System

**DOI:** 10.3390/ani9100753

**Published:** 2019-09-30

**Authors:** Miaomiao Xin, Pavlina Vechtova, Anna Shaliutina-Kolesova, Zoltan Fussy, Dmitry Loginov, Borys Dzyuba, Otomar Linhart, Serhii Boryshpolets, Marek Rodina, Ping Li, Yana Loginova, Jan Sterba

**Affiliations:** 1 South Bohemian Research Center of Aquaculture and Biodiversity of Hydrocenoses, Research Institute of Fish Culture and Hydrobiology, Faculty of Fisheries and Protection of Waters, University of South Bohemia in Ceske Budejovice, Zatisi 728/II, 389 25 Vodnany, Czech Republic; kolesova@frov.jcu.cz (A.S.-K.); bdzyuba@frov.jcu.cz (B.D.); linhart@frov.jcu.cz (O.L.); sboryshpolets@frov.jcu.cz (S.B.); rodina@frov.jcu.cz (M.R.); liping2018@email.sdu.edu.cn (P.L.); 2 Sino-Czech Joint Laboratory of Fish Conservation and Biotechnology: Key Laboratory of Freshwater Biodiversity Conservation, Ministry of Agriculture of China, Yangtze River Fisheries Research Institute, Chinese Academy of Fishery Sciences, Wuhan 430223, China; 3 Institute of Chemistry, Faculty of Science, University of South Bohemia in Ceske Budejovice, Branisovska 1760, 37005 Ceske Budejovice, Czech Republic; p.vechtova@seznam.cz (P.V.); zoltan.fussy@gmail.com (Z.F.); dloginov@prf.jcu.cz (D.L.); yloginova@jcu.cz (Y.L.); sterbaj@prf.jcu.cz (J.S.); 4 Biology Centre of Academy of Sciences of the Czech Republic, Institute of Parasitology, Branisovska 31, 37005 Ceske Budejovice, Czech Republic; 5 Marine College, Shandong University (Weihai), Weihai 264209, Shandong, China

**Keywords:** kidney, sperm, testes, transcriptome, Wolffian duct

## Abstract

**Simple Summary:**

Sturgeon is an ancient and unique fish species. Most of sturgeon are listed as critically endangered species due to habitat alteration and overharvesting. Study of sturgeon reproductive system and sperm is important for aquaculture and conservation programs. Transferrin is recognized as a multiple task protein, positively correlated with spermatogenesis and sperm quality. Thus, we tried to detect transferrin in spermiating and out-of-spawning sterlet reproductive organs and sperm. Two transferrin genes, serotransferrin and melanotransferrin, have been identified in reproductive organs of sterlet males. The serotransferrin was expressed higher in reproductive organs of spermiating than out-of-spawning sterlet males. Furthermore, transferrin was detected in sterlet seminal plasma. This information contributes to the existing information on the variability of transferrin proteins and the potential role of transferrin in chondrostean fishes.

**Abstract:**

Transferrins are a superfamily of iron-binding proteins and are recognized as multifunctional proteins. In the present study, transcriptomic and proteomic methods were used to identify transferrins in the reproductive organs and sperm of out-of-spawning and spermiating sterlet (*Acipenser ruthenus*) males. The results showed that seven transferrin transcripts were identified in the transcriptome of sterlet, and these transcripts were qualified as two different transferrin genes, serotransferrin and melanotransferrin, with several isoforms present for serotransferrin. The relative abundance of serotransferrin isoforms was higher in the kidneys and Wolffian ducts in the spermiating males compared to out-of-spawning males. In addition, transferrin was immunodetected in sterlet seminal plasma, but not in sterlet spermatozoa extract. Mass spectrometry identification of transferrin in seminal plasma but not in spermatozoa corroborates immunodetection. The identification of transferrin in the reproductive organs and seminal plasma of sterlet in this study provides the potential function of transferrin during sturgeon male reproduction.

## 1. Introduction

Transferrins are a superfamily of iron-binding proteins that play an essential role in ion transport from sites of absorption and storage to iron-requiring cells, and in the regulation of iron levels in biological fluids. It is a major serum protein, composed of two structurally homologous lobes (termed the N-lobe and the C-lobe), both containing a high-affinity ferric iron-binding site [1]. Iron-binding properties of transferrins implicate their involvement in nutritional immunity by acting as a bacteriostatic agent, as they impose an iron-restricted environment on bacteria, effectively impeding their respiration [2]. Besides binding iron, transferrins are recognized as having multiple roles in the protection of spermatozoa from bacterial actions [3,4] and oxidative damage [5], as well as cadmium toxicity [6,7]. 

Previously, transferrin was identified as a major protein in common carp (*Cyprinus carpio*) seminal plasma with a concentration of 330 to 340 µg/mL [8] and its unique properties in terms of iron ions-binding were demonstrated. Seminal fluid transferrin levels are used as an indicator of sperm quality, since it is positively correlated with sperm concentration and motility characteristics [9,10]. In addition, transferrins were also identified in liver, testis, head kidney, spleen, and efferent ducts of cyprinids and salmonids [8,11,12]. Testicular transferrin is synthesized and secreted mostly by Sertoli cells, which are regulated by peptide and steroid hormones known to be related to spermatogenesis [13,14]. The phylogenetic context of transferrin sequence was reported in several fish species, such as zebrafish [15], Atlantic cod [16], medaka [17]. and salmonids [12,18,19]. 

Traditionally, fish sperm for artificial propagation are collected so that urine contamination is avoided. Urine is hypotonic to semen in teleostean fish and considered to induce motility prematurely, thereby reducing sperm quality [20]. In sturgeon, urine from kidney was shown to trigger the last maturation step of spermatozoa, when they obtain the capacity to initiate motility [21,22] and thus fertilize [23]. Unspecified high molecular weight substances in the mixture of urine and seminal plasma were suggested to be essential in sturgeon spermatozoa maturation [22]. Since transferrins have been identified as major proteins in fish seminal plasma and positively correlated with sperm quality [8,10], we hypothesized that transferrins may be present in sturgeon seminal plasma and play an important role in sturgeon sperm maturation. 

The sterlet, *Acipenser ruthenus,* is widely used as a model fish among sturgeon to study artificial propagation and larviculture, as it has a relatively small body size and the shortest reproductive cycle in the family of Acipenseridae. Importantly, neither the presence of transferrin in the seminal fluid or spermatozoa, nor in male reproductive system, has been explored in sturgeon. Therefore, our study focused on (1) the confirmation of transferrin expression and identification of the various transferrin isoforms in the organs of the sterlet reproductive tract—the testes, kidney, and Wolffian duct; (2) the comparison of expression levels of transferrin genes in reproductive organs between spermiating and out-of-spawning sterlet males; (3) the identification of the transferrin proteins in sterlet seminal plasma and spermatozoa by mass spectrometry.

## 2. Materials and Methods 

### 2.1. Ethics Statement

All experiments were specifically approved by the Ethics Committee for the Protection of Animals in Research of the University of South Bohemia in Ceske Budejovice, Faculty of Fisheries and Protection of Waters, Vodnany, based on the EU-harmonized Animal Welfare Act of the Czech Republic (35086/2016-MZE-17214).

### 2.2. Fish and Sample Collection

The research was carried out at the facilities of the Genetic Fisheries Center of the Faculty of Fisheries and Protection of Waters, University of South Bohemia, Czech Republic. Sterlet males (age: 6–7 years, body weight: 2–3 kg) were used in experiments. Two groups of fish were arranged as follows: (1) Group of mature spermiating fish—specimen were caught from fish farming ponds in March (the start of natural spermiation season) and transferred to 4 m^3^ tanks with a closed water recirculation system, where the water temperature was gradually increased from 3 °C up to 15 °C by a 1 °C increment per day. This group was used for sperm sampling and for collection of reproductive organs for transcriptome sequencing. (2) Group of mature fish at out-of-spawning season—the group consisted of the males under nonspermiating conditions collected in August. This group was used for collection of reproductive organs for transcriptome sequencing only.

Spermiating sterlet males from group 1 were stimulated after a 5-day period of staying at 15 °C without feeding. Before stripping (24 h), spermiation was induced by an intramuscular injection of carp pituitary dissolved in 0.9% (*w*/*v*) NaCl solution at a dose of 4 mg/kg body weight. Sperm was collected from the urogenital tract using a 4 mm catheter connected to a 20 mL plastic syringe with gentle abdominal massage. Then, the sperm from three males with motility higher than 80% were placed on ice under aerobic conditions before experimentation. Immediately after sperm collection, the fish from group 1 and 2 were euthanized by striking the cranium, followed by exsanguination. After euthanasia, kidney, testes, and Wolffian ducts were identified according to protocol by Dzyuba et al. [24] and extracted for transcriptome sequencing from males of group 1 and 2.

### 2.3. Transcriptomics 

Total RNA of kidney, testes, and Wolffian duct of three mature spermiating males and three out-of-spawning mature males was isolated using RNA Blue (Top-Bio, Prague, Czech Republic) according to manufacturer’s instructions. The concentration of RNA was measured using NanoPhotometer (Implen, Munich, Germany) and the quality of RNA was determined using 2100 Bioanalyzer with RNA 6000 Nano kit (Agilent Technologies, Santa Clara, CA, USA). cDNA and sequencing library construction and sequencing of RNA samples were performed by the GeneCore facility at EMBL, Heidelberg, Germany. In brief, cDNA synthesis and sequencing library construction were carried out using NextSeq 500/550 High Output kit v2.5 (85 Cycles) (Illumina, San Diego, CA, USA). Sequencing was performed using the NextSeq 500 sequencing platform. Sequencing adapters were trimmed off and short reads were removed using Trimmomatic [25] with the following quality trimming parameters: ILLUMINACLIP:2:40:15 MINLEN:36. Trimmed reads were assembled using Trinity 2.6.6 [26]. The quality of the assembly was evaluated in Quast-2.3 [27] and the completeness of the transcriptome was verified with BUSCO 3.0.2 [28] against the Ensembl database of Actinopterygii orthologs (date of download 2018-11-12). Transcriptomic data were searched with known transferrins of related species as queries (namely *Lepisosteus oculatus* XP_015217073.1, *Ictalurus punctatus* ACN42672.1, *Danio rerio* DAA01798.1, *Salmo trutta* ACC55225.1, *Siniperca chuatsi* AJP74817.1, *Salmo salar* AAA18838.1, *Oreochromis niloticus* ABB70391.1) using the tblastn algorithm of BLAST (ver 2.2.31+) with default parameters. The relative abundances of transferrin isoforms in the three organs of both spermiating and out-of-spawning males were calculated using RSEM software package [29] implemented within the Trinity Transcript Quantification pipeline. Gene expression across all samples was evaluated as described in Huang et al. [30] using the EdgeR 3.24.3 package of Bioconductor [31]. In brief, the expression matrix of transcript abundances among different stages and organs was calculated and weakly expressed transcripts were removed (transcripts with less than 1 read per million in less than 3 libraries). All libraries were then normalized using TMM (trimmed mean of M-values) cross-sample normalization. 

The phylogenetic relationship of the putative *A. ruthenus* transferrin isoforms with known transferrin sequences of other Osteichthyes fish species (Chondrostei, Actinopterygii) and selected tetrapoda species (Sarcopterygii, Tetrapoda) available in GenBank and Ensembl database (last accessed on 2019-09-21) (see Figure 2 for accession numbers) was based on MAFFT v7.313 alignments constructed with L-INS-i local alignment optimization [32] and inferred using maximum likelihood estimation in IQtree v.1.6.8) [33] with ultrafast bootstrap approximation of maximum likelihood (ML) nodes support (-bb 1000) in two rounds of guide/final tree inference posterior mean site frequency model (PMSF) with LG+F+G and LG+C20+F+G as models, respectively [34]. The tree was rooted using the transferrin sequences of the outgroup *Callorhinchus milii* (downloaded on 2019-09-20) from Chondrichthyes class (Holocephali, Chondrichthyes). 

### 2.4. Protein Samples Preparation

The sperm samples collected from three mature spermiating males were centrifuged individually at 300× *g* at 4 °C for 30 min followed by 10 min at 13,000× *g* at 4 °C. The supernatant (seminal plasma) was carefully collected and stored at −80 °C. The pellets (spermatozoa) were collected and suspended in protein extraction buffer (8 M urea, 2 M thiourea, 4% CHAPS, 10% *w*/*v* isopropanol, 0.1% *w*/*v* Triton X-100, 100 mM dithiothreitol) containing HALT protease and phosphatase inhibitors (Thermo Fisher Scientific, Waltham, MA, USA). The suspended samples were vortexed continuously for 1 h, and subsequently centrifuged for 10 min at 13,000× *g* at 20 °C. The supernatant containing proteins was collected separately and stored at −80 °C. Bicinchoninic acid assay (Thermo Fisher Scientific, Waltham, MA, USA) was used to determine the protein concentration in the samples of seminal plasma and spermatozoa, using the Infinite M200 photometer (Tecan, Männedorf, Switzerland). 

### 2.5. D-PAGE Separation

Spermatozoa and seminal plasma proteins were analyzed by 2D electrophoresis. Isoelectric focusing was performed using ReadyStrip IPG strips pH 3–10, 17 cm length for spermatozoa; and 7 cm for seminal plasma (both Bio-Rad, Hercules, CA, USA) using the IEF100 instrument (Hoefer Holliston, MA, USA). A total of 800 μg of spermatozoa protein and 100 μg of seminal plasma protein were precipitated and dissolved in 300 and 125 μL rehydration buffer (7 M urea, 2 M thiourea, 2% CHAPS, 40 mM dithiothreitol, 0.2% Pharmalytes, 0.02% bromophenol blue), respectively, and used for IPG strip rehydration overnight at room temperature (RT). The conditions for isoelectric focusing (IEF) of 17 cm strips were: 250 V for 1.5 h (constant), 500 V for 3 h (constant), 1000 V for 6 h (gradient), 4000 V 2.5 h (gradient), and 4000 V for 6 h (constant); for 7 cm strips: 250 V for 30 min (constant), 1000 V for 1 h (gradient), 4000 V 2 h (gradient), and 4000 V for 2 h (constant) at 20 °C. After IEF, IPG strips were equilibrated with a buffer containing 6 M urea, 20% glycerol, 2% SDS, 0.375 M Tris–HCl pH 8.8, and 2% dithiothreitol for 20 min, followed by a second step with a similar solution containing 2.5% iodoacetamide instead of dithiothreitol for another 20 min. The strips were laid onto 12% SDS-PAGE gels and sealed with 0.5% agarose in SDS-PAGE running buffer. Spectra BR protein marker (Thermo Fisher Scientific, Waltham, MA, USA) and prestained Protein Marker VI (Applichem, Darmstadt, Germany) were used to estimate the molecular weight of the protein spots in spermatozoa and seminal plasma, respectively. After separation, proteins were fixed by fixative solution (50% ethanol, 2% phosphoric acid) and stained with Coomassie Brilliant Blue stain G-250 [35]. The images of gels were captured and digitalized using G:BOX Chemi XX6 system (Syngene, Cambridge, UK).

### 2.6. Western Blot Analysis

After 2D electrophoresis, proteins were electrically transferred for 1 h at 20 V on polyvinylidene difluoride (PVDF) membranes (7.5 cm × 8.5 cm) by semi-dry transfer in TRANS-BLOT SD (Bio-Rad). After the transfer, the membranes were incubated in the blocking solution [5% skimmed milk in phosphate-buffered saline with 0.05% *v*/*v* Tween-20 (PBS-T)] for 1 h at RT. After blocking, the membranes were washed three times with PBS-T for 6 min and incubated with the HPLC-purified primary anti-transferrin antibodies (1:200) diluted in blocking solution for 1 h at RT. Membranes were then washed and incubated with alkaline phosphatase-conjugated anti-rabbit antibodies produced in goat (1:1000 in blocking solution) (Vector Laboratories, Burlingame, USA; AP-1000) for 1 h at RT. The membranes were washed in PBS-T and developed in AP developing buffer (100 mM Tris, 100 mM NaCl, 10 mM MgCl_2_, pH 9.5) mixed with BCIP/NTP (3% NBT in 70% *N*,*N*-dimethylformamide, 1.5% BCIP in *N*,*N*-dimethylformamide) without shaking. 

### 2.7. In-Gel Digestion and MALDI-MS and MS/MS Analysis

All the seminal plasma protein CBB-stained spots and spermatozoa protein spots visualized in the Western blot analysis were excited from the corresponding SDS-PAGE gels, individually trypsin digested, and subjected to MALDI-MS and MS/MS. In-gel digestion followed by peptide extraction were carried out according to a protocol by Shevchenko et al. [36]. Protein digests were dissolved in 0.1% (*v*/*v*) trifluoroacetic acid (TFA) and separated using a microgradient device as described in [37]. Briefly, a microcolumn was wetted with 80% acetonitrile (ACN)/0.1% TFA and equilibrated with 0.1% TFA. After the loading, samples were eluted using a gradient of ACN from 2% to 50% (total volume of 12 μL) supplemented with 0.1% TFA directly onto an MSP AnchorChip^TM^ 384 target plate (Bruker Daltonics, Billerica, MA, USA) in 0.5 μL aliquots and mixed with 0.5 μL of α-cyano-4-hydroxycinnamic acid matrix solution.

MS and MS/MS spectra were acquired using an Autoflex Speed mass spectrometer controlled by flexControl 3.4 software (both Bruker Daltonics). WarpLC 1.3 (Bruker Daltonics, Billerica, MA, USA) was used for automatic measurement of microgradient MALDI fractions. Spectra were processed using flexAnalysis 3.4 and ProteinScape 3.1 software (Bruker Daltonics, Billerica, MA, USA).

Database searches were performed using the program Mascot Server 2.4 (Matrix Science, Boston, MA, USA) against in-house prepared databases containing either protein sequences from fishes belonging to the order Cypriniformes downloaded from National Center for Biotechnology Information (NCBI) database (accessed 2018-06-05; 533,418 sequences) or transferrin sequences obtained from the transcriptomic analysis in this study. Parameters used for database search were as follows: enzyme specificity—trypsin, two missed cleavages were allowed; fixed modifications—carbamidomethylation of cysteine; variable modifications—N-terminal protein acetylation and methionine oxidation. Precursor ion tolerance was set at 50 ppm, whereas the mass tolerance for MS/MS fragment ions was set at 0.5 Da. 

## 3. Results

### 3.1. Identification of Transferrin Transcripts in Sterlet

We sequenced RNA samples isolated from kidney, testes, and Wolffian ducts of both spermiating and out-of-spawning developmental stages of *Acipenser ruthenus*, each from three male individuals. We received 431 million (M) reads for spermiating males and 539 M reads for out-of-spawning males (150 M reads for out-of-spawning kidney, 225 M reads for out-of-spawning testes, 164 M reads for out-of-spawning Wolffian duct, 167 M reads for spermiating kidney, 153 M reads for spermiating testes, and 111 M reads for spermiating Wolffian duct). 

Trinity assembler produced an assembly of 455,101 contigs (sequence length of the shortest contig at 50% of the total genome length N50 = 1918 as computed by quast) and BUSCO found 81.5% of complete orthologs from the provided Actinopterygian conserved reference set. Raw reads were mapped to the assembly per sample, followed by abundance estimation using the RSEM alignment-based method. The matrix of read counts was built and weakly expressed transcripts were filtered out, which removed 357,718 out of 455,101 transcripts. Finally, the raw counts were normalized across libraries using TMM to correct for different library sizes and to obtain unified expression levels across individuals. TMM normalization is done using a scaling factor that is calculated for each library and each factor should be close to 1 [38]. Two outlier libraries (SM2K and SM2WD) were excluded from the relative abundance calculation as their small size was a result of technical variation during the sample processing and sequencing procedure (Table S1); their inclusion in later analyses would falsely underestimate abundance values. 

**Figure 1:**
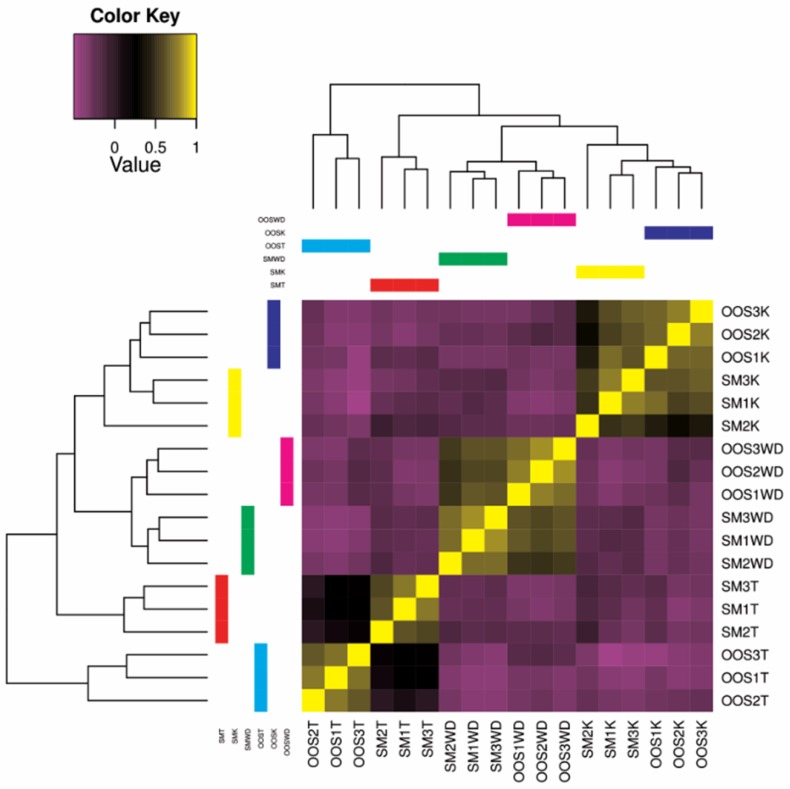
Sample correlation matrix heatmap, showing the degree of similarity of differentially expressed transcript clusters between each sample. SM: spermiating; OOS: out-of-spawning; K: kidney; WD: Wolffian duct; T: testes.

The differential expression among the studied sterlet samples, as calculated by edgeR, is shown as a sample correlation heatmap matrix (Figure 1). The expression profile between the out-of-spawning and spermiating individuals is more similar in the kidney and Wolffian duct compared to a rather variable profile in testes.

In total, seven transcripts homologous to known fish transferrin sequences were identified in the transcriptome of *Acipenser ruthenus*. The transcripts were qualified by Trinity assembler as two different transferrin genes with several isoforms present for serotransferrin and one isoform for melanotransferrin. All the isoforms of these two genes were translated to protein sequence based on the open reading frames (ORFs) identified by homology. The annotated sequences were deposited in GenBank (see Table 1 for accession numbers). For sterlet serotransferrin, isoform 3 encodes a complete ORF, while the remaining five isoforms encode incomplete ORFs truncated at either 5′- (isoforms 1 and 5) or 3′-termini (isoforms 2, 4 and 6). Sterlet melanotransferrin transcript encodes a complete ORF (Table 1).

**Figure 2:**
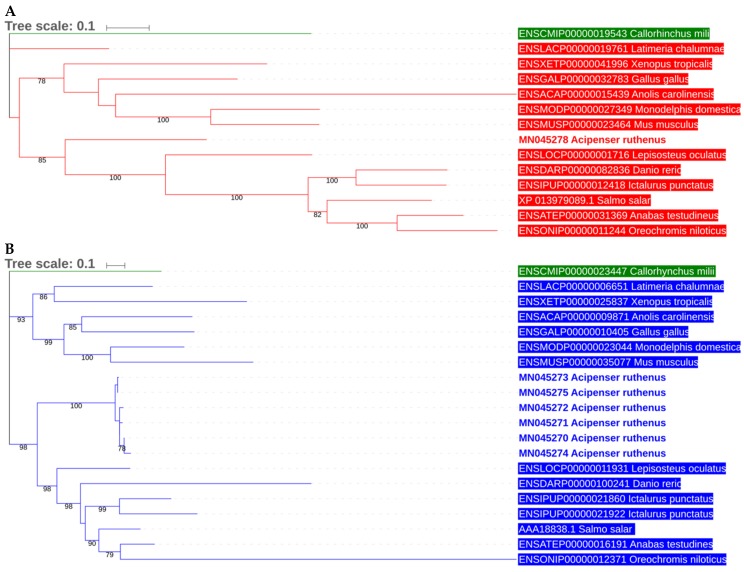
Phylogram showing the relationship of putative *A. ruthenus* transferrin isoforms with other transferrin sequences of related fish species and selected tetrapods. **Panel A**, melanotransferrin; **panel B**, serotransferrin. The ML phylogram was inferred by IQ-TREE with the guide/final tree inference (PMSF) strategy (see Material and Methods). The tree was rooted using sequences from *Callorhinchus milii* (Chondrichthyes, Chimaeriformes).

The phylogenetic relationship of the putative serotransferrin isoforms and melanotransferrin with known transferrin sequences of other related bony fish species (Osteichthyes) was calculated using the maximum likelihood estimation (Figure 2A,B). Serotransferrin isoforms and melanotransferrin formed discrete, well-supported clusters with the serotransferrin and melanotransferrin genes from other fishes. There was no apparently accelerated evolution of fish transferrins compared to Sarcopterygii (tetrapods), consistent with previous observations [39].

### 3.2. Expression of Transferrin in Reproductive Organs of Sterlet Males

**Figure 3:**
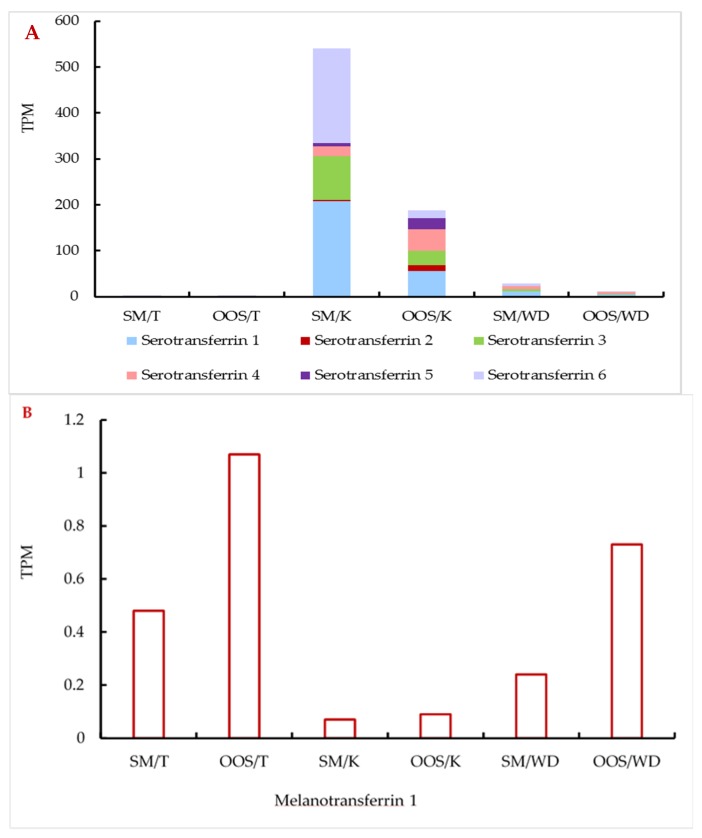
Relative abundances (TPM) of serotransferrin (**A**) and melanotransferrin (**B**) transcripts in the individual organs of both the mature and out-of-spawning sterlet males. (**A**) also shows the ratio of individual serotransferrin isoforms in each sample. SM: spermiating; OOS: out-of-spawning; K: kidney; WD: Wolffian duct; T: testes.

Relative abundances of the individual transferrin transcripts and their isoforms in the studied reproductive organs were compared. Overall, serotransferrin showed a much higher expression in all the studied organs (Figure 3A) compared to melanotransferrin (Figure 3B), especially in the kidney (ca three orders of magnitude). Serotransferrin showed approximately three-times higher expression in the kidney and Wolffian duct in the spermiating fish (Figure 3A), while the expression of melanotransferrin was increased in the out-of-spawning males (Figure 3B).

These trends were similar for most of the isoforms in all the studied samples with few exceptions—serotransferrin isoforms 2, 4, and 5 were found to be more expressed in some of the organs of out-of-spawning males (Table 2).

### 3.3. 2-DE Analysis and Western Blot of Sterlet Seminal Plasma and Spermatozoa

**Figure 4:**
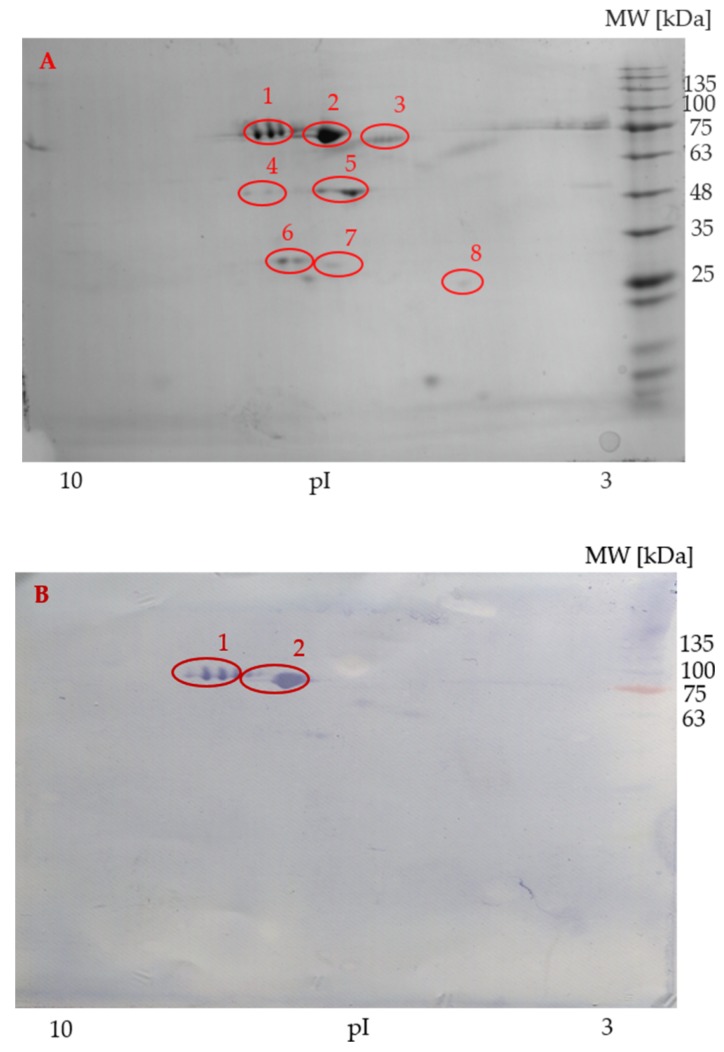
Representative sterlet seminal plasma protein profile (**A**) visualized by two-dimensional gel electrophoresis. Immunoblot of sterlet seminal plasma with specific antibodies (**B**). Circles show the identified protein spots, two of which were reactive and subjected to MALDI-TOF/TOF MS. Molecular weight marker is on the right. pI = Isoelectric points.

Two-dimensional IEF/SDS-PAGE and immunolabelling with anti-transferrin antibodies were performed on sterlet seminal plasma and spermatozoa samples. The protein profile of seminal plasma is shown in Figure 4A. Four strong, specific signals belonging to two proteofroms were detected on the PVDF membrane (Figure 4B). The isoelectric points (pI) of the reacted protein spots on seminal plasma PVDF membrane were in the range of 6 to 7.5 and molecular weights reached 75 kDa. 

**Figure 5:**
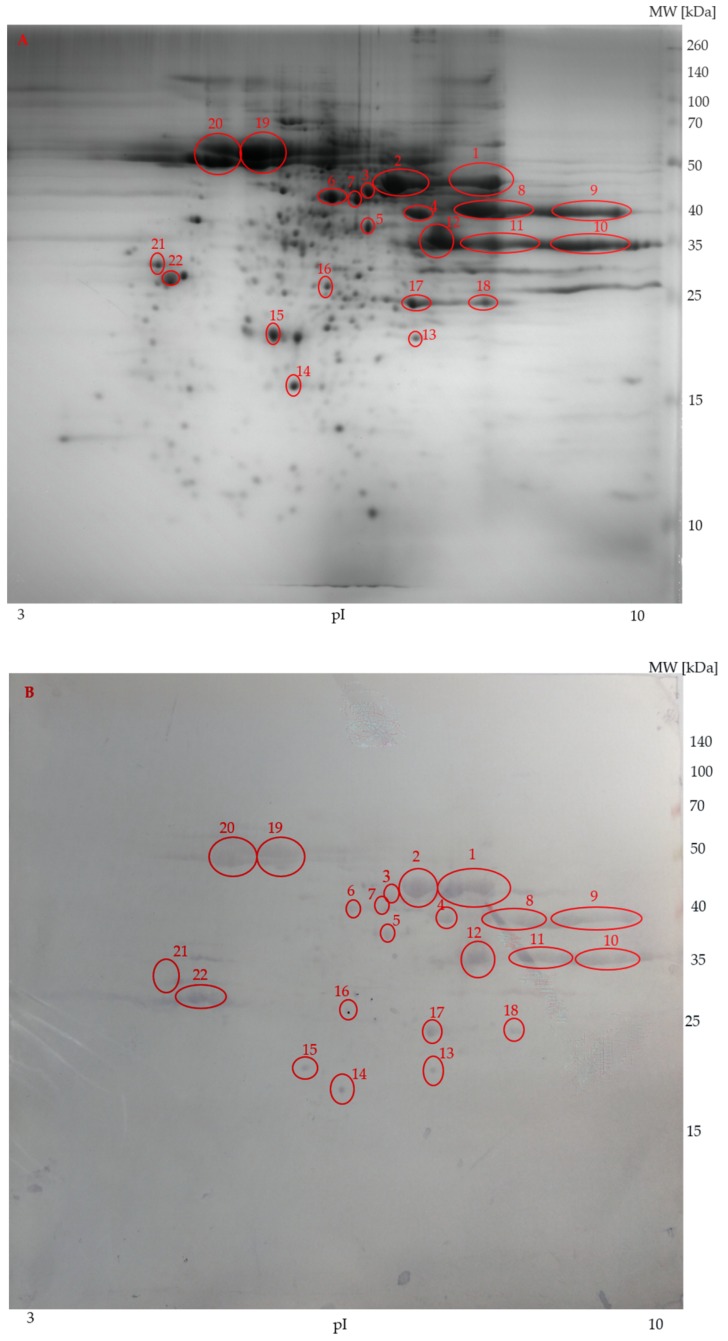
Representative spermatozoa protein profiles (**A**) of sterlet by two-dimensional gel electrophoresis. Western blot reaction of sterlet spermatozoa (**B**). Circles show the reacted protein spots subjected to MALDI-TOF/TOF MS. Molecular weight marker is on the right. pI = Isoelectric points.

On the other hand, anti-transferrin antibody reacted with proteins of sterlet spermatozoa (Figure 5A) only nonspecifically (Figure 5B). Thus, the presence of transferrins in spermatozoa was not confirmed.

### 3.4. Identification of Transferrin by MALDI-MS and MS/MS

In seminal plasma, eight protein spots from the 2-DE/SDS-PAGE gels (Figure 4A) were individually subjected to mass spectrometry. Transferrins were identified in spot 1, which also reacted positively on the PVDF membrane. Another identified protein was hypothetical protein cypCar_00035444 (triosephosphate isomerase), identified in spot 8. No proteins were identified in spots 2–7. The source organism, accession number, mascot score, molecular mass, number of identified peptides, and sequence coverage of two transferrin proteins and hypothetical protein cypCar_00035444 (triosephosphate isomerase) in sterlet seminal plasma are given in Table 3. 

Immunoblotting of the spermatozoa protein with anti-transferrin antibodies resulted only in nonspecific antibody binding. The recognized protein spots were analyzed by MS to verify the presence of transferrin in the recognized spots. Proteins were identified in five of them (Table 4). These proteins were enolase in spots 1 and 2; tubulin in spot 19, 20, and 22. Transferrin was not detected in any of the analyzed spots using either the publicly available database or the in-house database produced from our transcriptomic data. No proteins could be identified in spots 3–18 and 21. Mass data from these unidentified protein spots were compared with protein sequences deposited in public databases, but no positive sequence coverage for these protein spots was detected. The more detailed results about source organism, accession number, mascot score, molecular mass, number of identified peptides, and sequence coverage of these identified proteins in sterlet spermatozoa are given in Table 4.

## 4. Discussion

Previously, transferrin was found in the kidney and seminal plasma of mammals [40,41] and fish, such as cyprinids, salmonids, and Miichthys [8,10,11,12,42], with the function of iron binding and transportation. To our knowledge, this study is the first to systematically identify transferrin genes in different reproductive organs of sterlet and to assess their expression profiles in both spermiating and out-of-spawning males. Moreover, this is the first study that demonstrated the presence of transferrin protein in the seminal plasma of sturgeon.

Transferrins are ion binding proteins that belong to a family of glycoproteins, including serotransferrin (often referred to simply as transferrin) in blood, ovotransferrin (conalbumin) in bird egg whites, lactoferrin in mammalian biological fluids, and melanotransferrin in membranes of melanocarcinoma cells [15,43,44]. Here, we identified two different transferrin genes, serotransferrin and melanotransferrin, with six and one isoforms, respectively, in the reproductive organs (kidney, Wolffian duct, and testes) of sterlet. Serotransferrin plays a crucial role in iron metabolism and maintenance through binding or transport of iron in kidney cells [45]. This is consistent with histological investigations that have shown the presence of hemopoietic cells almost exclusively in the kidneys of tench and the high specific activity of hemin-iron in the kidney compared to spleen and erythrocytes [46]. We, as well, observed high expression of serotransferrin in the kidney, especially in spermiating sterlet males. Overall, serotransferrin expression was lower in the Wolffian duct and testes in sterlet males, compared to the kidney, though its expression was higher in Wolffian duct and testes of spermiating males compared to out-of-spawning males. The exact function of transferrin in Wolffian duct of sturgeon had not been proposed to date because the Wolffian duct is missing in other fish species. Based on our results, we suggest that Wolffian duct’s transferrin might contribute to the maturation and protection of sterlet spermatozoa during their passage [5], although the exact function needs further investigation.

Melanotransferrin is a minor player in iron metabolism. It was originally identified at high levels in melanoma cells, having a role in melanoma cell proliferation and tumorigenesis [47,48]. Kawamoto et al. [49] showed that melanotransferrin is highly expressed in cartilage and chondrocytes, but is barely detectable in the other tissues of male Japanese White rabbits. Similarly, we detected lower expression of melanotransferrin gene in the kidney, the Wolffian duct, and testes of both spermiating and out-of-spawning sterlet males. While serotransferrin isoforms are more expressed in spermiating males compared to out-of-spawning males in sterlet, melanotransferrin shows higher expression in out-of-spawning males. Other studies, too, report very low expression of melanotransferrin in most adult tissues; whereas significant levels of melanotransferrin protein were reported in developing tissues (such as fetal colon, small intestine, or in the umbilical cord and heart) and in neoplastic tissues [50,51]. The expression of melanotransferrin in the small intestine was supposed to act as an enhancer of the neonate’s absorption of iron from mother’s milk, which only contains low concentrations of iron [51]. Thus, the higher expression of melanotransferrin in the organs of out-of-spawning sterlet males may be due to the importance of iron absorption, especially in the Wolffian duct and the testes, when serotransferrin is not highly expressed. In addition, one melanotransferrin gene has been reported in numerous vertebrate and invertebrate species [52,53,54], except for the four different melanotransferrin genes in the transcriptome of the sea cucumber *Holothuria glaberrima* [55]. We only identified one melanotransferrin gene in the transcriptome of sterlet reproductive organs, which plays a fundamental role for the exploration and current literature of melanotransferrin.

Transferrins were shown to have undergone a complex evolutionary process. Vertebrate transferrin was vastly different from that of invertebrates, and the transferrins of the ancestors of aquatic and terrestrial organisms were exposed to different selection pressures [42]. From our analyses, it is obvious that two transferrin genes, serotransferrin and melanotransferrin, form discrete, well-supported clusters and that the phylogeny of both genes corresponds to the overall classification of bony fishes [56].

We identified peptides of two serotransferrin isoforms 3 and 4 in sterlet seminal plasma, but not in spermatozoa. This is consistent with the view that urine contributes in a major way to the final composition of seminal plasma and that the actual source of seminal transferrin is the kidney where its production is the largest. Both transcriptomic and mass spectrometry data support this notion (Table 2, Table 3). Transferrin has also been identified in human, carp, and rainbow trout seminal plasma [6,8,9,11,57]. Transferrin level is an index of gonadal function in men [9], with decreased levels observed in obstructive azoospermic men compared to healthy individuals [58]. In common carp, transferrin was identified as a major seminal plasma protein and plays an important role in protection of carp spermatozoa from microbes, heavy metal toxicity, and oxidative attack [2,8,59]. Similarly, transferrin appeared as a major protein in sterlet seminal plasma (Figure 4). While the function of transferrin in sterlet seminal plasma remains unexplored, it is reasonable to assume that transferrin plays an important role in protection of spermatozoa of sterlet as in other metazoans. Transferrin was immunodetected in several salmonids’ seminal plasm, but there was a weak detection in rainbow trout spermatozoa [12]. Furthermore, immunohistochemical analysis revealed the presence of transferrin in cytoplasm of spermatogonia and secondary spermatocytes, but absence in cysts containing spermatids [12]. Likewise, transferrin was detected in rat testis, proacrosome, and nuclear cap of developing spermatids, but almost not at all in mature spermatozoa; this indicates the requirement of high levels of iron during the early stages of spermatid development and the importance of transferrin for supporting spermatogenesis [13,60,61]. In the present study, transferrin failed to be identified in mature sterlet spermatozoa. Further investigation will be needed to understand the precise role of transferrin during sturgeon spermatogenesis and sperm maturation.

In contrast to other cyprinid fish, sturgeon sperm after differentiation in testes passes through the kidney to mix with hypotonic urine and then through Wolffian ducts where they are stored [24]. The exposure of testicular sperm to urine triggers physiological maturation in the final steps of spermatogenesis, leading to the acquisition of the potential for sperm motility and fertilization [22,23]. Besides hypotony, the presence of unknown high molecular weight substances (>10 kDa) and calcium ions in urine and seminal fluids was suggested to be a prerequisite for spermatozoa maturation [22]. In the present study, high expression of transferrin was detected in both seminal plasma and kidney, especially in the spermiating male sterlet. Thus, we propose that transferrin may play an important role for sterlet sperm maturation and protection [5].

## 5. Conclusions

Significant variations of transferrin expression in kidney, Wolffian duct, and testes was detected between spermiating and out-of-spawning sterlet males, implying that transferrins may play a general role in male reproduction in fish. Considering the transferrins as major proteins identified in sterlet seminal plasma, the importance of physiological and protective roles of this protein for sturgeon sperm was subsequently suggested. The identification of different isoforms of serotransferrin and melanotransferrin genes contributes to the existing information on the variability of transferrin proteins in chondrostean fishes.

## Figures and Tables

**Table 1:** List of identified genes and their isoforms in the sterlet together with their NCBI accession numbers. * incomplete ORFs.

Transcript Name	NCBI Accession Number	Length (nt)	ORF
Serotransferrin isoform 1	MN045270	465	1–465 *
Serotransferrin isoform 2	MN045271	1790	285–1790 *
Serotransferrin isoform 3	MN045272	2651	285–1907
Serotransferrin isoform 4	MN045273	1212	187–1212 *
Serotransferrin isoform 5	MN045274	425	1–425 *
Serotransferrin isoform 6	MN045275	1310	285–1310 *
Melanotransferrin isoform 1	MN045278	4514	263–2467

**Table 2:** Relative abundances (TPM) of the sterlet serotransferrin and melanotransferrin isoforms in the studies samples.

	Serotransferrin						Melanotransferrin
Isoforms	1	2	3	4	5	6	1
SM T	0.92	0.23	0.44	0.36	0.33	0.66	0.48
OOS T	0.68	0.12	0.32	0.69	0.08	0.16	1.07
SM K	207.37	3.66	94.61	22.17	7.34	205.45	0.07
OOS K	55.46	13.02	31.93	46.99	23.07	17.46	0.09
SM WD	11.17	0.01	5.31	6.54	0.09	5.57	0.24
OOS WD	4.09	0.24	1.85	3.61	0.54	1.13	0.73

SM: spermiating; OOS: out-of-spawning; K: kidney; WD: Wolffian duct; T: testes.

**Table 3:** List of proteins identified in the seminal plasma of sterlet (*Acipenser ruthenus*).

Spot No.	Accession No. ^(a)^	Description ^(b)^	Organism	MW, kDa/pI ^(c)^	Score ^(d)^	No. of Peptides ^(e)^	SC, % ^(f)^
1	MN045272	serotransferrin isoform 3	*Acipenser ruthenus*	58.50/6.78	482.87	11	24.80
	MN045273	serotransferrin isoform 4	*Acipenser ruthenus*	37.40/6.79	367.31	9	25.70
8	gi|966652723	hypothetical protein cypCar_00035444 (Triosephosphate isomerase)	*Cyprinus carpio*	26.50/5.13	113.60	2	8.70

(a) This accession number refers to the sequence retrieved from the NCBInr protein database. (b) A description of the respective protein accession. Protein homologs were assigned to unknown proteins via BLAST search. (c) A theoretical molecular weight and pI values. (d) The probability-based score value resulting from the MS/MS search. (e) Number of identified peptides. (f) Sequence coverage (SC) calculated from the peptide MS/MS data.

**Table 4:** List of proteins identified in the spermatozoa of sterlet (*Acipenser ruthenus*).

Spot No.	Accession No. ^(a)^	Description ^(b)^	Organism	MW, kDa/pI ^(c)^	Score ^(d)^	No. of Peptides ^(e)^	SC, % ^(f)^
1	gi|1025390473	PREDICTED: beta-enolase	*Sinocyclocheilus rhinocerous*	47.40/6.58	513.09	10	24.90
	gi|966672540	hypothetical protein cypCar_00005974, partial (enolase-like)	*Cyprinus carpio*	47.30/4.81	338.33	5	18.20
2	gi|1025390473	PREDICTED: beta-enolase	*Sinocyclocheilus rhinocerous*	47.40/6.58	355.54	11	21.00
	gi|966672540	hypothetical protein cypCar_00005974, partial (enolase-like)	*Cyprinus carpio*	47.30/4.81	321.81	6	14.30
19	gi|82414773	UNVERIFIED_ORG: zgc:123298	*Danio rerio*	49.90/5.01	847.58	13	35.80
	gi|45709036	Tuba1 protein	*Danio rerio*	50.10/4.94	808.62	14	40.40
	gi|47940377	Zgc:55461	*Danio rerio*	49.80/4.79	634.28	11	31.00
	gi|468861133	tubulin alpha 1-like protein 2	*Hypophthalmichthys molitrix*	49.20/4.91	628.46	12	37.70
	gi|295314924	tubulin beta 1	*Hypophthalmichthys molitrix*	49.70/4.79	613.81	11	27.60
	gi|966714399	hypothetical protein cypCar_00019490 (tubulin)	*Cyprinus carpio*	49.80/5.05	555.40	9	25.40
20	gi|295314924	tubulin beta 1	*Hypophthalmichthys molitrix*	49.70/4.79	740.86	13	42.70
	gi|1025170763	PREDICTED: tubulin beta chain-like	*Sinocyclocheilus rhinocerous*	49.60/4.81	658.89	10	35.10
	gi|966703762	hypothetical protein cypCar_00027299 (tubulin)	*Cyprinus carpio*	50.40/4.72	582.05	9	32.30
	gi|1101617233	PREDICTED: tubulin beta chain-like isoform X1	*Cyprinus carpio*	55.90/5.33	553.66	9	26.30
22	gi|1101525613	PREDICTED: tubulin beta-1 chain-like	*Cyprinus carpio*	49.70/4.75	87.37	2	6.10

(a) This accession number refers to the sequence retrieved from the NCBInr protein database. (b) A description of the respective protein accession. Protein homologs were assigned to unknown proteins via BLAST search. (c) A theoretical molecular weight and pI values. (d) The probability-based score value resulting from the MS/MS search. (e) Number of identified peptides. (f) Sequence coverage (SC) calculated from the peptide MS/MS data.

## Supplementary Materials

The following are available online at https://www.mdpi.com/2076-2615/9/10/753/s1, Table S1: Summary of library mapping statistics and abundance cross sample normalization.
