# Supplementary material for: Transferrin Identification in Sterlet (Acipenser ruthenus) Reproductive System
Source: Animals (Basel). 2019 Sep 30;9(10):753. doi: 10.3390/ani9100753 (PMC6826671; doi:10.3390/ani9100753)
Supplement: Supplementary file 1 [file animals-09-00753-s001.pdf]

Table 1. Summary of library mapping statistics and abundance cross sample normalization.

		Mapping statistics				Expression statistics		
**Library**	**Number of reads**	**Mapped reads**	**% of mapped reads**	**Unmapped reads**	**% of unmapped reads**	**TMM normalized read counts**	**TMM normalized read counts after filtering of lowly abundant transcripts**	**Library scaling factor**
SMT 1	53440623	48,260,135	90.31	5180488	9.69	45532222.1	43931385.2	1.0718479
SMT 2	49,897,547	45,957,118	92.10	3940429	7.9	44144732.7	43056902	1.0657851
SMT 3	49,973,877	45,398,147	90.84	4575730	9.16	43173900.6	41872086.4	1.0272566
SMK 1	61,348,977	55,335,962	90.20	6013015	9.8	52299986	50523526.8	1.1217139
SMK 2*	835,132	756,039	90.53	79093	9.47	718694.5	693121.6	2.0784005
SMK 3	104,393,182	94,651,335	90.67	9741847	9.33	90094797	87161788.5	1.0371642
SMWD 1	57,863,993	52,630,494	90.96	5233499	9.04	50216209.8	48537253.2	1.2986722
SMWD 2*	5,543,592	5,030,786	90.75	512806	9.25	4797482.4	4610817.6	2.0751077
SMWD 3	47,657,325	43,204,051	90.66	4453274	9.34	41152468.7	39733203.8	1.3079599
OOST 1	90,521,683	86,910,223	96.01	3611460	3.99	85113072.8	84250538.9	0.2288785
OOST 2	73,695,504	69,264,005	93.99	4431499	6.01	66919440.1	62848835.4	0.4543562
OOST 3	60,606,686	55,846,772	92.15	4759914	7.85	53573703.5	51511616.8	0.9037775
OOSK 1	47,726,402	43,384,531	90.90	4341871	9.1	41320497	40344048.7	0.9253682
OOSK 2	50,656,839	46,122,711	91.05	4534128	8.95	44075304.2	43367512.1	0.7101671
OOSK 3	51,857,246	47,275,479	91.16	4581767	8.84	45035961.5	44063103.9	0.8360825
OOSWD 1	46,834,319	42,579,130	90.91	4255189	9.09	40627646	39176641.6	1.2827232
OOSWD 2	47,991,255	43,546,243	90.74	4445012	9.26	41487984.2	40124367.7	1.2468346
OOSWD 3	69,094,158	62,469,774	90.41	6624384	9.59	59429709.9	57278743.9	1.2105859

SM: spermiating; OOS: out-of-spawning; K: kidney; WD: Wolffian duct; T: testes.
